# Heat adaptation of phage T7 under an extended genetic code

**DOI:** 10.1093/ve/veab100

**Published:** 2021-12-01

**Authors:** Austin W Cole, Steven D Tran, Andrew D Ellington

**Affiliations:** Department of Molecular Biosciences, Institute for Cellular and Molecular Biology, University of Texas, 2500 Speedway Ave., MBB 3.424, Austin, TX 78712, USA; Department of Molecular Biosciences, Institute for Cellular and Molecular Biology, University of Texas, 2500 Speedway Ave., MBB 3.424, Austin, TX 78712, USA; Department of Molecular Biosciences, Institute for Cellular and Molecular Biology, University of Texas, 2500 Speedway Ave., MBB 3.424, Austin, TX 78712, USA

**Keywords:** experimental evolution, T7 bacteriophage, engineered codon table, thermal adaptation

## Abstract

While bacteriophages have previously been used as a model system to understand thermal adaptation, most adapted genomes observed to date contain very few modifications and cover a limited temperature range. Here, we set out to investigate genome adaptation to thermal stress by adapting six populations of T7 bacteriophage virions to increasingly stringent heat challenges. Further, we provided three of the phage populations’ access to a new genetic code in which Amber codons could be read as selenocysteine, potentially allowing the formation of more stable selenide-containing bonds. Phage virions responded to the thermal challenges with a greater than 10°C increase in heat tolerance and fixed highly reproducible patterns of non-synonymous substitutions and genome deletions. Most fixed mutations mapped to either the tail complex or to the three internal virion proteins that form a pore across the *E. coli* cell membrane during DNA injection. However, few global changes in Amber codon usage were observed, with only one natural Amber codon being lost. These results reinforce a model in which adaptation to thermal stress proceeds via the cumulative fixation of a small set of highly adaptive substitutions and that adaptation to new genetic codes proceeds only slowly, even with the possibility of potential phenotypic advantages.

## 1. Introduction

Bacteriophages have proven to be an excellent vehicle for understanding laboratory evolution (Burch and Chao 1999; Turner and Chao 1999; Crill, Wichman, and Bull 2000; Montville et al. 2005; McBride, Brandon Ogbunugafor, and Turner 2008). By studying phage, it has proven possible to better understand how complex systems—composed of multiple different protein and nucleic acid parts that further interact with the complex metabolism of their cellular hosts—traverse diverse or congruent evolutionary paths when presented with environmental challenges. We and others have attempted to understand not only how wild-type phage systems but also modifications of these systems, such as changes in the phage’s organization, gene content, or even genetic code impact the paths and outcomes of directed evolution (Bull, Molineux, and Wilke 2012; Cecchini et al. 2013; Willemsen et al. 2018). In particular, earlier reports have shown that phage can readily adapt their genome in response to engineered codon tables, for example, unnatural amino acids have been shown to sometimes be positively selected in bacteriophage T7 (Hammerling et al. 2014), and ssRNA phage Qβ readily recovered fitness following adaptation to a novel codon table that ambiguously coded for both tryptophan and 6-fluorotryptophan (Bacher, Bull, and Ellington 2003).

However, there have to date been few experiments that tried to alter the complex systems (the phages) in a rational way so that it could better evolutionarily adapt to new environmental challenges. In particular, we wished to better understand how phage might utilize an altered genetic code to potentially assist with adaptation to progressively higher temperature challenges. To this end, bacteriophage T7 was cultivated on an engineered *E. coli* strain that lacked all Amber codons and their associated release factor (Lajoie et al. 2013) and that also contained machinery for the broad insertion of twenty-first amino acid selenocysteine (Thyer et al. 2015, 2018). As opposed to past experiments in which an altered genetic code and evolutionary task were relatively unrelated, we hoped that stable diselenide bonds (that can form even in the reducing environment of the cell) might arise and thereby lead to the selection of thermostable phage variants.

## 2. Selection design and implementation

T7 bacteriophage virions have previously been selected for virion stability at 60°C, but no adaptive response was observed (Heineman and Brown 2012). In contrast, a 5.4 kb ssDNA phage ΦX174 adapting to growth at an elevated temperature reproducibly fixed several ‘big-benefit’ mutations (Bull, Badgett, and Wichman 2000). This suggests that thermally adaptive mutations within the T7 genome may be infrequent, epistatic, or individually of little effect (Heineman and Brown 2012).

We thus further hypothesized that the ability to form covalent intra- or intermolecular crosslinks would lead to more frequent or better thermal adaptation. Disulfide bridges have previously been shown to stabilize proteins and are over-represented in coding sequences of thermophiles that carry a oxidoreductase to help with cytosolic disulfide maturation (Creighton 1988; Beeby et al. 2005). Diselenide bridges have an even lower redox potential than disulfide bridges (−381 mV compared to −180 mV) and can form intramolecular covalent bonds in the otherwise reducing *E. coli* cytoplasm (Walewska et al. 2009; Thyer et al. 2018). The opportunity to incorporate selenocysteine might therefore provide phage an advantageous adaptive strategy for individual proteins, and in turn the phage as a whole, to become more fit at higher temperatures. We had previously engineered a strain of *E. coli* that could unambiguously code for selenocysteine at all Amber codons (hereafter referred to as ‘seleno-adapted’), starting from an *E. coli* strain that had been edited to remove all 321 Amber stop codons and the relevant release factor RF1, hereafter referred to as ‘Amberless’ (Lajoie et al. 2013; Thyer et al. 2015, 2018).

**Figure 1.**
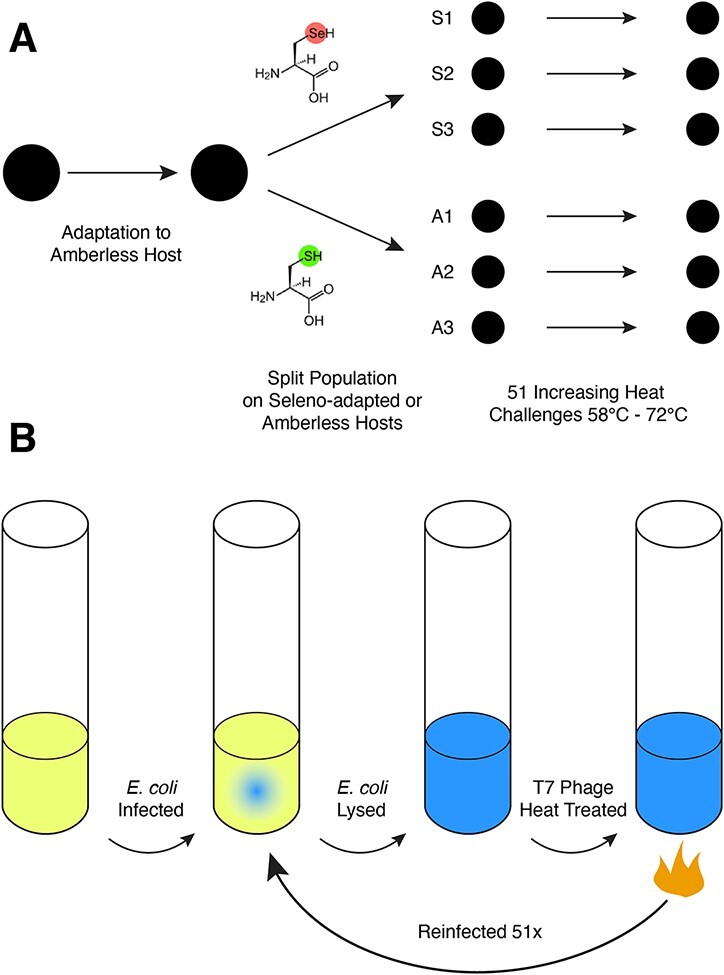
(A) An overview of the evolution experiment. One phage population was passaged twelve times on the Amberless host. This population was then split into six populations. Three of these populations were grown on the seleno-adapted host (S1–S3) and three were grown on the Amberless host (A1–A3). Virions in all six populations were subjected to iteratively more stringent heat treatments. (B) A graphical presentation of the heat selection procedure. *E. coli* hosts were infected with phage populations and lysed. Phage virions were then heat-treated for 1 h and surviving phage were reinfected on a fresh host culture.

We set up an experiment to passage bacteriophage T7 on both strains and treat virions with increasingly higher temperatures to determine whether the phage would take advantage of the underlying biochemical potential of adopting an unnatural amino acid (Fig. 1). To disambiguate the molecular signatures of phage evolution on these two strains, T7 phage were initially serially passaged on the parental Amberless host for 75 generations during which time the population fixed a previously reported deletion (see **Results**) and three novel non-synonymous substitutions (see **Discussion**). Then virion thermotolerance was evolved on either Amberless *E. coli* (A1–A3) in the absence of selenium (prohibiting selenocysteine production) or on the seleno-adapted strain (S1–S3) in the presence of selenium. In addition, the evolution experiments described herein maintained a large population size to ensure that selection overwhelmed drift, thereby bolstering the likelihood that infrequent mutations of large effect would sweep to fixation and lead to stabilized virions. Roughly 10^10^ virions in 20 ml of lysate were heat-treated and then given an opportunity to infect susceptible hosts. Since the Amber reversion rate in T7 is measured to be roughly 10^−6^ (Drake et al. 1998), every single step nucleotide substitution is expected to be present in about 10,000 individuals during each thermal challenge. This was important because the heat shocks applied in this experiment iteratively killed at least 99.9 per cent of phage in the evolving population. In order to accelerate evolution and avoid clonal interference, we also allowed infected host cultures to fully lyse prior to each round of selection, which has previously been used in T7 Phage evolution experiments to promote coinfection and recombination (Muller 1964; Springman et al. 2012).

It is worth noting that exposure to heat might alter the frequency and type of mutations recovered. In dsDNA phage T4, heating greatly elevates the absolute frequency of G–C to A–T transitions and to a lesser degree elevates G–C to C–G transversions, as measured by Amber reversions (Baltz, Bingham, and Drake 1976; Bingham et al. 1976; Drake and Baltz 1976). If this were to also prove true with T7 bacteriophage, a much higher mutational load could further limit the survival of individual beneficial variants in each round. However, given that a previous report successfully elevated the mutational load in T7 populations by two orders of magnitude using a chemical mutagen, N-methyl-N′-nitro-N-nitrosoguanidine, and did not induce population collapse through mutational meltdown (Springman et al. 2010), it is unlikely that the heat attributable mutation burden here would approach lethal mutagenesis.

## 3. Results

### 3.1 Adaptive response to a thermal challenge

**Figure 2.**
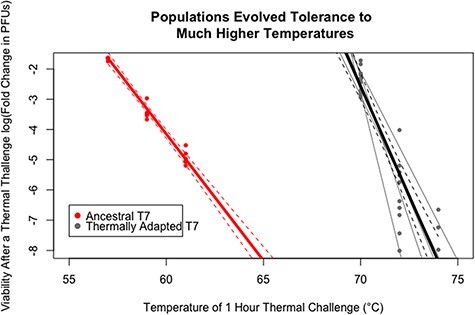
Phage populations evolved tolerance to a thermal challenge. The fraction of phage that could form a plaque after a heat challenge increased in all evolved populations over the course of adaptation. Red dots depict the infectivity profile of ancestral phage in response to increasing thermal stress while grey dots depict the infectivity profile of evolved phage in response to thermal stress. Each grey line corresponds to a single adapted population’s profile and the black line corresponds to the aggregate profile of adapted phage. Population A3 is not depicted because although 5 × 10^−4^ pfus were observed at 70°C, the response at higher temperatures was unresolved. Dotted lines represent 95 per cent confidence intervals about linear regressions for ancestral and adapted responses.

First, lysates of T7 phage were prepared on an Amberless host and the virions were subjected to thermal challenges at 57°C, 59°C, and 61°C. A regression of residual infectivity after these challenges indicated that roughly one in a thousand wild-type T7 virions were expected to be infective after an hour at 58°C (Fig. 2). Challenges were started at this temperature to avoid strong population bottlenecks and Muller’s Ratchet effects (Chao 1990).

For a given challenge temperature, each of the six adapting populations was subjected to three cycles of heat treatment and infection to lysis on their respective host (Fig. 1), that is, a phage population infected a host, was heat-treated, infected, heat-treated, infected, and heat-treated at a single challenge temperature. After the three successive challenges, the six populations were plated and plaques were counted to estimate overall survival. If more than 10^5^ virions/ml—or roughly 0.01 per cent of the population—retained infectivity across all populations, the challenge temperature was increased by 1°C. This experimental setup syncs the adaptive response of the population to further environmental challenges. Phage populations passed this criterion 14 times over the course of 51 heat challenges while failing to meet this criterion three times, thereby requiring an additional three challenges at the failed challenge temperatures in order to sufficiently adapt. Ultimately, the challenge was increased to 1 h at 72°C, at which point the experiment was terminated.

Following thermal adaptation, a high frequency (>10^−4^) of virions in all six populations retained infectivity after 1 h thermal challenge at 70°C. A small but detectable fraction (10^−7^) of virions from A1, A2, and S3 was even viable after being challenged for 1 h at 74°C. While no difference was observed in the adaptive response between T7 evolving on the seleno-adapted or Amberless strains as measured by the fraction of a population retaining infectivity after a thermal challenge (*P* > 0.1 by anova), a large response was observed between all evolved populations and the ancestor (*P* < 10^−10^ by anova).

### 3.2 Sequence analysis

The six heat adapted populations (A1–A3, S1–S3) and the pre-adapted population were harvested after infecting and lysing one liter of their exponentially growing Amberless or seleno-adapted *E. coli* host. Purified DNA was sequenced as MiSeq 2 × 300 paired-end reads at the University of Idaho sequencing facility, and then nucleotide substitutions, duplications, and deletions were identified after mapping to a reference T7 genome, NCBI ref. NC_001604.1, using BreSeq (**Supplementary Materials**) (Deatherage and Barrick 2014; Hammerling et al. 2014). Only mutations that were observed in >80 per cent of reads mapping to the reference genome are presented in the results.

### 3.3 Non-synonymous substitutions and duplications

The six evolved populations each fixed between 7 and 12 non-synonymous point mutations during laboratory adaptation, and 12 of the 30 distinct amino acid substitutions were present in independent populations (Table 1). These substitutions were for the most part localized in the structural genes 8, 11, and 12 that encode the tail complex, and in genes 14, 15, and 16 that encode internal virion proteins responsible for pore formation (Lupo et al. 2015).

**Table 1.** Several nucleotide substitutions and one duplication fixed independently during laboratory adaptation.

Nucleotide position	Nucleotide mutation	Populations fixed	Coding effects of amino acid substitution
18,406	G→A	A1, A2, S1, S3	Gene 6: Amber to ochre stop; Gene 6.3: A5S
18,419	T→C	S2	Gene 6.3: L9P
19,721	G→A	A2	Gene 7.3: E63K
20,544	G→A	A1, A2, A3, S2, S3	Gene 8: G102E
21,265	A→C	A3, S1, S2, S3	Gene 8: K342N
21,276	A→G	A2	Gene 8: D346G
21,279	C→T	A1	Gene 8: A347V
21,285	A→C	S2, S3	Gene 8: E349A
21,734	A→G	S3	Gene 8: M499V
21,736	G→T	A1	Gene 8: M499I
24,161	A→C	A2, A3	Gene 10B: *58S now reads: 58SLA61*
24,238	A→G	A2, A3, S1, S3	Gene 11: Y4C
24,475	A→G	A3	Gene 11: D83G
26,746	G→T	A3	Gene 12: E635D
26,753	G→A	A1	Gene 12: G638S
26,756	A→G	S1	Gene 12: K639E
27,769	12 bp duplication	S1	Gene 14: 13GA now reads 13GAISGA
27,783	G→A	A1, A3, S2, S3	Gene 14: G19D
27,932	T→C	A2, A3, S1, S3	Gene 14: S69P
28,691	A→C	A1, A2, S1, S2	Gene 15: K123Q
28,692	A→G	A3	Gene 15: K123R
29,304	A→C	S2	Gene 15: E327A
29,486	G→A	S2	Gene 15: D388N
29,697	C→A	A2	Gene 15: A458E
30,869	G→A	S3	Gene 16: G92D
32,079	A→C	S3	Gene 16: K495N
34,975	A→G	A2, S2	Gene 17: T118A
34,982	C→T	A1	Gene 17: T120I
35,366	C→A	A1, A3	Gene 17: S248Y
36,054	T→G	A1	Gene 17: S477R
37,346	T→C	A2, A3, S1, S3	Gene 18.5: *144Q now reads 144QEIK*

The substitutions K342N, D346G, A347V, and E349A in gene 8 map to the inside of the ejection pore, where pressurized genomic DNA is positioned at the tail exit gated by gene 12. Two other substitutions at the C-terminus of gene 8 (M499V and M499I) map to the crown at the inside of the virion core where several copies of the internal virion proteins that form the pore penetrating the *E. coli* membrane and genomic DNA reside until pore formation is triggered by *E. coli* receptor binding (Guo et al. 2014). Thus, one consequence of heat adaptation may have been to alter the thermodynamics or kinetics of DNA injection into the host (see also **Discussion**).

### 3.4 Genome deletions

A previously reported 1.4 kb deletion spanning non-essential genes emerged during pre-adaptation to Amberless *E. coli*. The deletion starts at the C-terminus of gene 0.3, extends through the entirety of gene 0.4, 0.6A, and 0.6B, and stops at the C-terminus of protein kinase gene 0.7. A series of additional genomic deletions that were between 769 and 1,019 bp long and that spanned the non-essential genes 4.5 and 4.7 evolved in parallel across four of the heat tolerant populations of phage, and notably across the S1–S3 populations (Table 2). Deletions often emerge in response to heat adaptation and have been reported several times (Ritchie and Malcolm 1970; Studier 1973). These deletions occurred between intergenic regions that shared seven to nine bp of homology. In population A1, a two bp frameshift deletion was also observed at H38 of the phage lambda-like lysis protein, gene 18.5.

**Table 2.** Large genomic deletions fixed independently during laboratory adaptation. Deletions covering genes 4.5–4.7 fixed during adaptation in response to a thermal challenge while the deletion covering genes 0.3 to the protein kinase fixed during adaptation to the Amberless host (a locus where adaptive deletions have been reported in previous studies) (Cunningham et al. 1997; Hammerling et al. 2014). Precisely replicated deletions in populations S2 and S3 are possibly attributable to contamination; however, the two populations share only four SNPs, while a further fourteen SNPs distinguish them.

Position	Length	Homology	Lines	Gene
1,253	1,489 bp	GAGGAAGTCG	WT*	C-terminus of gene 0.3 to the N-terminus of the protein kinase
13,278	1,019 bp	TTCTTGA	A3	C-terminus gene 4.2 through N-terminus of gene 4.7
13,569	770 bp	TAATCAA	S1	All of gene 4.5 through all of gene 4.7
13,579	769 bp	AGGAGAAA	S2, S3	All of gene 4.5 through all of gene 4.7

## 4. Discussion

### 4.1 Heat adaptation

Former heat adaptation experiments either adapted the virus to a host grown at an elevated temperature (Dowell 1980; Bull, Badgett, and Wichman 2000; Crill, Wichman, and Bull 2000; Kichler and Bull 2001; Knies et al. 2006; McBride, Brandon Ogbunugafor, and Turner 2008; Knies, Kingsolver, and Burch 2009; Dessau et al. 2012), or they challenged virions in isolation with an elevated temperature (Ritchie and Malcolm 1970; Kadowaki et al. 1987; Cox et al. 2010; Singhal et al. 2017). In both approaches, a small collection of highly adaptive substitutions often reproducibly fix during adaptation. For example, when single-stranded DNA bacteriophage ΦX174 was selected for growth as measured by plaque formation on an *E. coli* host at 45°C, only three unique substitutions were each found to overcome heat inhibition and enable plaque formation at the elevated temperature (Bull, Badgett, and Wichman 2000). These three ‘big-benefit’ substitutions were localized in the coat protein and an internal scaffolding protein, suggesting that heat inhibited virion assembly and stability more than phage replication and infection. In a separate example, 15 populations of lytic, double-stranded RNA bacteriophage Φ6 virions were subjected to 30 heat challenges in which the temperature was increased from 45°C to 50°C either all at once or more gradually (Singhal et al. 2017). No correlation between the rate of temperature change and final infectivity after a thermal challenge at 50°C was observed, but the researchers identified a conserved set of six parallel (independently evolved) substitutions in the P5 lysis protein and the P5 outer shell protein (the only genes sequenced), with the average population fixing only 2.1 substitutions in the two genes.

The experiments described herein are distinct from those carried out previously by starting adaptation at the edge of the wild-type virion’s heat tolerance, with the first heat challenge being at 58°C and the last at 72°C. By consistently assessing the survival of the population prior to new challenges, a greater than 10°C improvement in the highest temperature tolerated was obtained, while comparable previous experiments with Φ6 led to an adaptive response of less than 5°C. As was the case with previous experiments, there were a number of unique, fixed substitutions that were found in parallel across all populations. Specifically, 61 fixed point mutations occurred at 30 unique sites across all populations and 41 of these fixed substitutions occurred in parallel at 12 sites. The probability of observing such parallel substitutions through purely random mutagenesis without selection and fixation is exceedingly small (Beletskii et al. 2000). Amazingly, no synonymous substitutions were observed in any of the evolved genomes. Taken together, these results strongly suggest the phage genomes were evolving in response to the thermal challenges. The average adapted T7 genome fixed 10.2 substitutions and shared 6.8 of these substitutions with another phage.

During initial adaptation to the Amberless host it was expected that populations would fix mutations at Amber stop codons to reestablish wild-type translational termination; however, the three substitutions that fixed during the early adaptation were all coding substitutions within the DNA polymerase (gene 5), the internal virion protein C (gene 15), and the tail fiber (gene 17). The collection of unique mutations observed in this experiment included only a single substitution away from an Amber stop codon at gene 6, which had also been reported in previous studies (Hammerling et al. 2014). Evolved genomes retained five to six intact Amber codons that presumably allowed for protein synthesis to extend through normally untranslated intergenic regions, suggesting either that genes terminating in Amber stop codons were non-essential in this context or that the unstructured C-terminal additions to these proteins did not deprecate function sufficiently for selection to act on. This is especially interesting given that a single nucleotide change would have allowed the adoption of a functional stop codon (Ochre). Given that it seems likely that at least some fraction of the proteins were extended beyond their encoded termini, it is possible that these extensions provided fodder for adaptation in their own right.

### 4.2 Sequence changes

#### 4.2.1 Tail complex substitutions

**Figure 3.**
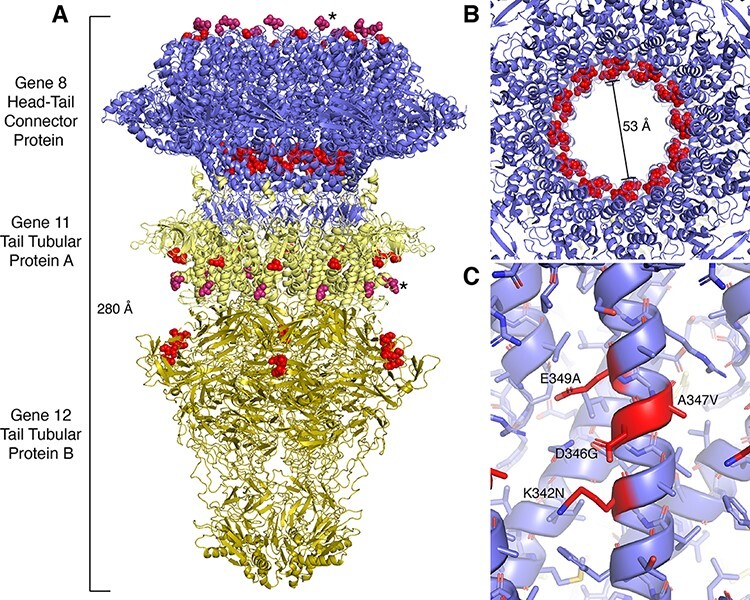
Several amino acid substitutions that fixed during laboratory adaptation can be mapped to a resolved structure of the T7 tail complex. (A) The entirety of the tail complex is 280 Å long and comprised of dodecameric gene 8, dodecameric gene 11, and hexameric gene 12. Variable, resolved residues are marked as red spheres while variable residues that lie on unresolved regions were flagged by the (*) and salmon spheres at their nearest resolved residue. (B) The gene 8 aperture is 53 Å wide in the open conformation, and four variable sites lie at a stem alpha helix. (C) Several substitutions along a stem alpha helix in gene 8 reorient the steric packing of the alpha-helical barrel while preserving dense hydrogen bonding network.

The structures of tail genes 8, 11, and 12 were recently resolved as a fiberless tail complex of dodecamers (genes 8 and 11) coordinated with a hexamer (gene 12) absent the rest of the T7 virion (PDB: 6R21) (Cuervo et al. 2019). This structure suggests a mechanistic model for the retention and ejection of the 40 kb linear phage genome. Several substitutions colocalized in gene 8 (Fig. 3). Substitutions at four residues (K342N, D346G, A347V, and E349A) all resided along an internal alpha helix that connects the protein dodecamers and interacts with genomic DNA. This eight residue region was the only narrow region (<10 residue window) that fixed substitutions across all six population, and these four sites accounted for eight of the sixty-one independently fixed SNPs accumulated during the heat adaptation experiment, strongly indicating that these substitutions may have fixed in response either to heat selection or in response to genomic deletions.

One substitution (K342N) along the stem of the head–tail connector protein appears to disrupt a stable cation–pi interaction with F338 or a salt bridge with D346 and was independently fixed four times. The asparagine should still be able to participate in favorable amide-pi interactions (Ottiger et al. 2009; Singh and Das 2015) with both F338 and a salt bridge (Kumar and Nussinov 1999) with D346 while simultaneously minimizing steric interactions between adjacent alpha helices that form the alpha-helical barrel. The loss of the positively charged amino acid at the alpha-helical junction may also allow for tighter packing of alpha helices within this motif, which would be further buttressed by the A347V mutation.

#### 4.2.2 Capsid protein substitutions

Several studies have detailed biophysical mechanisms of virion stability that are related to the major capsid proteins (Lee et al. 2011; Stone et al. 2019). These stabilizing mechanisms include disulfide bonds within and between protein subunits of the capsid, interlaced loops between adjacent faces of the capsid protein, and charged surface interactions at the interface between capsid proteins (Bauer et al. 2015; Stone et al. 2019). Given that the stability of in a capsid is manifest in all 415 copies present in the virion (Guo et al. 2014), it might be expected that heat selection would favor stabilizing interactions between capsid proteins—in fact mutations stabilizing viral capsids have been identified during laboratory adaptation to heat in Type 1 poliovirus (Adeyemi et al. 2017) and in 5.6 kb ssDNA bacteriophage ID11 (Lee et al. 2011).

However, in the current experiments only two populations fixed capsid substitutions. The substitutions were in the frameshifted coat protein isoform gene 10B that comprises ∼5–10 per cent of the virion (Dunn, William Studier, and Gottesman 1983) and introduces a charged three-residue C-terminal extension (EIK) to the isoform. The C-terminus of gene 10B in mature virions is an unstructured extension at the surface of the capsid, and commercial phage display vectors taken advantage of this property by introducing heterologous proteins in place of the natural frameshifted extension (Danner and Belasco 2001). This termini is therefore ripe for mutability, and additional surface contacts could readily stabilize the coat proteins and their interactions.

#### 4.2.3 Genomic deletions

There is reason to think that a dsDNA bacteriophage encapsidated in a non-filamentous protein head complex is particularly susceptible to thermal challenge. The biophysical properties that wound, dehydrated dsDNA exert on a dsDNA T4 virion have been detailed by Bauer and Evilevitch, and may inform molecular adaptations observed here (Bauer and Evilevitch 2015). At increased temperatures premature dsDNA virion ejection is observed, and thus, it is possible that several large deletions (non-essential genes 4.5–4.7 via recombination potentiated by 7 or 8 bp of homology) were selected because a reduction of encapsidated DNA would concomitantly reduce pressure on the tail complex. These observations are also consistent with earlier reports that heat stable T7 isolates contained genome deletions (Ritchie and Malcolm 1970; Studier 1972). In addition, an adaptive 1.4 kb deletion at gene 0.3 was fixed during initial passaging on the Amberless host that had been reported earlier (Cunningham et al. 1997; Bull et al. 2003; Hammerling et al. 2014).

#### 4.2.4 Adaptation of heat-challenged phage to selenium

We expected that phage adaptation to a thermal challenge on the seleno-adapted host might favor changes to covalent bridge formation because: 1, stabilizing intra- and intermolecular disulfide bonds are over-represented in thermophiles, and 2, selenocysteine readily forms covalent diselenide bonds in the *E. coli* cytosol even in the absence of enzymatic cofactors (Thyer et al. 2015). While previous experiments in adapting *E. coli* to more broadly utilize selenocysteine in its genetic code revealed a number of genome modifications that could potentially be explained by the selective challenges imposed by this redox-active amino acid (Thyer et al. 2018), there were no selenocysteine-specific adaptations observed in the evolved phage populations. This may have been because heat adaptation was far more stringent than any challenges selenocysteine may have incrementally imposed (only 1:10^4^–1:10^8^ phage survived each thermal challenge), and the most available mechanisms for heat adaptation did not involve diselenide bond formation.

## 5. Conclusions

Bacteriophages are among the most abundant and diverse entities on earth (Thurber 2009; Paez-Espino et al. 2016; Al-Shayeb et al. 2020), and thermostable isolates have been found alongside their bacterial hosts in metagenomics studies in varied extreme environments including hot springs (Breitbart et al. 2004; Zablocki, Leonardo, and Trindade 2018), desert sands (Prigent et al. 2005), and recently deep sea hydrothermal vents (Castelán-Sánchez et al. 2019). While the features of fully resolved extremophilic phage genomes can be qualitatively compared to their close relatives (Zablocki, Leonardo, and Trindade 2018), the molecular signatures of thermal-adapted genomes cannot be easily separated from other fitness features (i.e. responses to host scarcity, population bottlenecks, or host–pathogen coevolution). Directed evolution studies can also be used to generate thermotolerant (treatment prior to infection (Ritchie and Malcolm 1970; Kadowaki et al. 1987; Cox et al. 2010; Singhal et al. 2017)) or thermostable (replicative co-cultures with host bacteria (Dowell 1980; Bull, Badgett, and Wichman 2000; Crill, Wichman, and Bull 2000; Kichler and Bull 2001; Knies et al. 2006; McBride, Brandon Ogbunugafor, and Turner 2008; Knies, Kingsolver, and Burch 2009; Dessau et al. 2012)) phage, but for the most part, populations in these previous studies fixed a small number of adaptive substitutions (fewer than three) and were not adapted to thermal stresses far outside the ancestral genome’s tolerance.

Here, we have explored the adaptation of a dsDNA phage in response to a progressive set of thermal challenges, ultimately obtaining particles that could survive at 74°C—a temperature that occurs in natural hot springs. Fewer than 0.1 per cent of the ancestral phage were infective after an hour of heat inactivation at 60°C, while the evolved phage retained 1 per cent infectivity after an hour of heat inactivation at 70°C. The six evolving populations adapted to the thermal stress via complex but consistent patterns that included modifications to the tail complex; some mutations in capsid proteins; and large, reproducible deletions that were not observed in other reports of heat adaptation in either ssDNA, ssRNA, or dsRNA bacteriophage.

Most interestingly, we attempted to provide the phage with a novel chemical path to the development of thermotolerance, evolution in the context of a novel genetic code in which Amber codons could be read as selenocysteine, an amino acid that can form novel intra- and intermolecular bonds that might have been expected to impart stability to phage variants. The phage apparently did not find the substitution to be detrimental, retaining all but one Amber stop codon, however, in agreement with earlier studies did not avail themselves of the evolution of new selenocytsteine-mediated covalent bonds (Thyer et al. 2018), possibly because two Amber mutations are required to form a stable diselenide bond. This may emphasize that neutral drift and the creation of epistatic potential with new amino acids might first be required before subjecting populations to more functional selections, such as the stringent heat selection we have devised herein.

## 6. Materials and methods

### 6.1 Strains and media

Wild-type T7 populations (GenBank AY264774) were grown on ‘Amberless’ *E. coli* whose genomes had been engineered to omit all Amber stop codons (Addgene catalog number 49018, MG1655 Δ(ybhB-bioAB)::zeoR ΔprfA) (Lajoie et al. 2013) or on an Amberless derivative that unambiguously codes for selenocysteine at the TAG codon (‘Amberless’ or ‘seleno-adapted’ strains). The seleno-adapted strain was laboratory adapted through 2,500 generations of evolution with dependence on selenocysteine incorporation enabled by a suppressor tRNA^sec^ variant and maintained by an engineered β-lactamase containing an essential diselenide bond (Thyer et al. 2018). Here, Amberless hosts were grown on LB supplemented with 33 µg/µl zeocin while seleno-adapted hosts were grown on LB supplemented with 25 μg/mL kanamycin, 50 μg/mL spectinomycin, 10 μM Na2SeO3, and 100 μg/mL carbenicillin as described previously (Thyer et al. 2018).

### 6.2 Early adaptation to Amberless *E. coli*

Prior to selection for thermal tolerance, wild-type T7 bacteriophages were adapted to the novel Amberless host by serial passaging at 37°C. In total 2 ml of exponentially growing Amberless *E. coli* at an OD of 0.1 was infected at a MOI of 0.01 and lysed. This phage was then serially transferred on exponentially growing Amberless *E. coli* by initially infecting two ml bacteria with 20 µl of lysate and then transferring 20 µl of infected culture to exponentially growing uninfected Amberless *E. coli* at an OD of 0.1 every 20 min for twelve passages. Infected bacteria in the twelfth passage were allowed to lyse, cell debris were cleared by filtration on a two-micron membrane, and purified T7 phages were archived as glycerol stocks at −80°C. The six evolving phage lineages were each seeded by an independent 2 µl aliquot from this glycerol stock.

### 6.3 Experimental evolution

In total, 5 ml of overnight cultures of Amberless or seleno-adapted hosts was diluted into 80 ml of LB and grown for 1 h at 37°C. After an hour, these bacteria were infected with 20 ml of heat-treated, crude lysate that had been chilled on ice for 5 min. The infected bacteria were allowed to lyse, usually over the course of 1 or 2 h, and this lysate was then incubated in a water bath at the challenge temperature for 1 h. This was repeated three times a day at a single temperature. Phage populations after the third heat treatment of the day were titrated in susceptible hosts and top agar to estimate their adaptive response as indicated by pfus/ml in heat-treated crude lysate. The temperature of the water bath and heat challenge was increased as the phage populations adapted to the thermal treatment—if more than 10^5^ pfus/ml could be counted for all evolving populations after the final heat challenge of the day, the temperature challenge for all populations was increased by 1°C. Heat challenges started at 58°C and increased to 72°C over the course of 51 treatments. Samples from each of the six co-evolving populations were preserved in glycerol stocks at −80°C after every third challenge.

### 6.4 Thermal resistance assay

The fraction of phage retaining infectivity after a heat treatment was estimated by dividing the pfu/ml of phage lysate after heat treatment for 1 h by the pfu/ml of phage lysate held on ice for 1 h. Fresh lysate for each population was prepared on their respective host prior to heat treatment. Subsamples of the lysate were incubated in an ice bath while separate 100 µl subsamples of the same phage populations were incubated across a gradient of temperatures in a thermocycler for an hour. After an hour, heat-treated and iced lysates were each titrated in top agar with their respective hosts and then plaques were counted the next day. Data points for regressions discriminating between evolved and wild-type phage populations were calculated by dividing the concentration of pfus for a population after 1 h heat treatment by the concentration of pfus for that same population held on ice for 1 h.

## Data availability

Breseq analysis of the sequencing data for populations A1–A3, S1–S3, and the pre-adapted phage are included in the **Supplementary Materials**.

## Supplementary data

Supplementary data is available at *Vevolu Journal* online.

## Supplementary Material

veab100_Supp
